# Association of Drug Rebates and Competition With Out-of-Pocket Coinsurance in Medicare Part D, 2014 to 2018

**DOI:** 10.1001/jamanetworkopen.2021.9030

**Published:** 2021-05-05

**Authors:** Darius Lakdawalla, Meng Li

**Affiliations:** 1 Leonard D. Schaeffer Center for Health Policy and Economics, University of Southern California, Los Angeles; 2 School of Pharmacy, University of Southern California, Los Angeles; 3 Sol Price School of Public Policy, University of Southern California, Los Angeles; 4 Department of Health Services Research, The University of Texas MD Anderson Cancer Center, Houston

## Abstract

**Importance:**

Prior research has documented the increase in prescription drug rebates and the coincident increase in out-of-pocket burden for patients paying coinsurance tied to list prices.

**Objective:**

To describe the out-of-pocket burden on patients with coinsurance and assess its association with pharmaceutical competition, which increases payers’ leverage to seek higher rebates.

**Design, Setting, and Participants:**

This retrospective cohort study used branded prescription drugs with US sales reported by publicly traded companies. The study included drugs with nonmissing, nonnegative rebates between 2014 and 2018 from SSR Health. Data analysis was conducted from June to December 2020.

**Exposures:**

Level of branded and generic competition and calendar year.

**Main Outcomes and Measures:**

Retail price markup (ie, the ratio of rebate to net price) paid by patients at the point of sale and effective out-of-pocket share (ie, coinsurance multiplied by list price divided by net price) of a standard Part D plan. Trends in these outcomes were examined and then stratified by degree of competition.

**Results:**

There were 3322 unique National Drug Codes in the analysis, representing 232 distinct molecules from 138 therapeutic classes in 34 disease areas. The ratio of rebate to net prices was higher and increased faster for drugs with branded and generic competitors (from 83% to 172%) than for drugs with only branded competitors (from 61% to 115%) and those without generic equivalents (from 33% to 49%). Hypothetical patients paying standard Part D coinsurance on drug list prices would have experienced an effective out-of-pocket share increase from 48% to 64% in the initial coverage phase, and from 10% to 13% in the catastrophic coverage phase between 2014 and 2018. In the coverage gap, the share increased from 92% in 2014 to 98% in 2016 and then decreased to 90% in 2018. Compared with drugs with no competition, effective out-of-pocket share paid by patients grew 50% faster for drugs with branded competitors and 100% faster for those with branded and generic competitors.

**Conclusions and Relevance:**

This study found substantial increases in cost-sharing burden for patients paying coinsurance on drug list prices between 2014 and 2018, especially in markets with more pharmaceutical competition. Payers passing rebates through to patients at the point of sale could restore the benefits of competition and rebates.

## Key Points

**Question:**

Is the out-of-pocket burden higher in more competitive drug classes when drug rebates do not reach patients at the point of sale?

**Findings:**

In this cohort study of 3322 unique National Drug Codes, mean drug list prices were 34% to 61% higher than mean net prices. For a hypothetical consumer paying standard Part D coinsurance on list prices, 2018 out-of-pocket share was 64% in the initial coverage phase, 13% in the catastrophic phase, and greater than 90% in the coverage gap; the patient share was higher for more competitive drug classes.

**Meaning:**

These findings suggest that the current rebate system places additional burden on patients exposed to list price–based coinsurance, particularly in competitive classes.

## Teaser

This study describes the out-of-pocket burden on patients with coinsurance and assesses its association with pharmaceutical competition.

## Introduction

Policy makers have begun to scrutinize rebates paid by drug manufacturers to pharmacy benefit managers and other third-party payers. Although manufacturers publicly announce what are known as list prices for their drugs, health insurers pay net prices (ie, list prices minus rebate payments). Prior research has documented an increase in rebate payments, which has coincided with an increase in list prices. For example, a 2020 study of 1335 US branded prescription drugs^1^ reported that between 2015 and 2018, the average rebate nearly doubled, and each additional dollar of rebate was associated with a $1.17 increase in list prices. An approximate doubling of rebates helped contain net prices despite rapid list price growth during this period.^2,3^

It is tempting to conclude that rapid list price growth is only a mirage because net prices represent what drugs actually cost. However, for some consumers, including many in Medicare Part D, list prices influence out-of-pocket cost for contractual reasons. Rebates are trade secrets disclosed only to the pharmaceutical manufacturers and the pharmacy benefit managers that negotiate them. To keep rebates confidential, these firms must also keep net prices confidential. To avoid disclosing net prices to consumers, insurers typically require consumers with coinsurance obligations to pay a percentage of the retail pharmacy price, not the confidential net price, as their coinsurance. Because list prices are within 5% of pharmacy drug acquisition costs on average, increases in list prices may cause retail pharmacy prices to increase and consumer out-of-pocket obligations to increase with them.^4^

On the other hand, the current rebate system has the advantage of making premiums decrease. Two of the largest pharmacy benefit managers—CVS and Express Scripts—reported that they return more than 90% of rebates to their commercial clients.^5,6^ Their clients, typically self-insured employers or insurance companies, could in principle choose to use these savings to restrain premium increases for their beneficiaries. In the context of Part D, for example, the Congressional Budget Office estimated that beneficiary premiums would increase if coinsurance were applied to net prices instead of list prices.^7^

On balance, rebates paid under the current system reduce premiums but increase out-of-pocket cost; they reduce the effective generosity of offered coverage because the share of net price paid out of pocket increases above the stated coinsurance rate. Such declines in generosity are likely to be most pronounced in the most competitive pharmaceutical markets. Greater competition provides purchasers with more negotiating leverage, leading to lower net prices, higher rebates, lower net drug costs, and thus lower premiums.^8^ Meanwhile, if patient coinsurance is associated with list prices, out-of-pocket obligations will increase more quickly than net prices, causing patients to pay an increasing share of the net price.

In this study, we quantified how trends in rebates and net prices might be associated with a reduction in the effective generosity of coinsurance-based plans. We then explored the extent to which these patterns varied with the competitiveness of different drug classes. Last, we used the Medicare Part D standard plan design to illustrate the consequences on hypothetical patients paying coinsurance on list prices.

## Methods

We followed the Strengthening the Reporting of Observational Studies in Epidemiology (STROBE) reporting guideline for cohort studies.^14^ This research was deemed exempt by the University of Southern California institutional review board. Consent was waived because no human participants were involved.

### Measuring the Generosity of Coverage

We measured insurance generosity by using the share of drug cost paid by the patient. We approximated the patient’s coinsurance obligation as *Coinsurance* × *List_d_*, where *Coinsurance* denotes the coinsurance rate and *List_d_* is the list price of drug *d*. (More specifically, coinsurance is applied to retail pharmacy prices. Because of data constraints, we used list prices to proxy for retail prices, recognizing that the 2 might not always coincide.) The underlying cost of the drug is the net price, defined as *Net_d_*. The patient’s share of the net price is given by
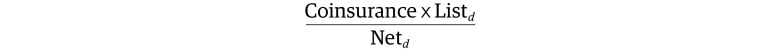
*Rebate_d_* was defined as the rebate paid on drug *d*, where *List_d_* =* Net_d_* *+* *Rebate_d_*. These definitions allow expression of the patient’s effective out-of-pocket share for drug *d* in benefit phase *b* as:
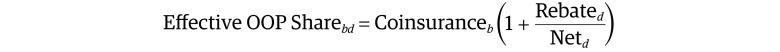
If coinsurance were paid on the basis of net prices, the effective out-of-pocket share would simply be the coinsurance rate. When coinsurance is paid on a price higher than the net, the effective out-of-pocket share exceeds the coinsurance rate. We defined 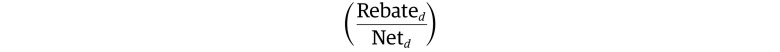
as the retail price markup that patients pay at the point of sale when paying coinsurance. For instance, if rebates are 75% of net prices, the retail price markup is 75% because patients pay prices 75% higher than true net prices.

### Data

To measure list and net prices, we used the Brand Rx net pricing tool (SSR Health), which provides quarterly list and estimated net prices per unit for approximately 1000 brand-name products for more than 100 companies.^9^ For every quarter in our data, SSR Health compiles product-level net revenues (gross revenues minus all manufacturer concessions, such as rebates, discounts, coupon cards and other patient assistance programs, and 340B discounts) reported by publicly traded pharmaceutical companies in their Securities and Exchange Commission filings. They collect product-level units sold to US end users from Symphony Health.^10^ They estimate net prices as net revenues divided by units sold. Data on units sold cover retail pharmacy, inpatient, outpatient, and other clinical settings. By finding the mean of their quarterly estimates for each of the relevant variables, we created annual mean list and net prices by National Drug Code and calculated the difference between list and net prices (ie, the rebate and other concessions made by manufacturers, the latter of which may include discounts, coupon cards, 340B discounts, and patient assistance programs as well as other similar price concessions) by National Drug Code. Codes with missing or negative rebates were excluded. Net revenues reported by manufacturers are ex-factory, whereas Symphony Health reports end-user volume. Rebates may be negative (net price > list price) if more products are shipped to the wholesaler than are distributed to patients. We conducted our analysis from 2014 to 2018 and express all prices in 2020 US dollars, using the Consumer Price Index to adjust for inflation.^11^

To measure the competitiveness of different drug classes, we gathered annual data from 2014 to 2018 on whether the National Drug Code was the only distinct molecule in its therapeutic class or whether it had generic equivalents in the market using information from First Databank.^12^ Last, we collected information on Medicare Part D drug use between 2014 and 2018 from the Medicare Part D Drug Spending Dashboard.^13^

### Statistical Analysis

We summarized list prices, net prices, rebates, and the ratios of rebate to net price (ie, the retail price markup) for drugs (ie, National Drug Codes) in our sample from 2014 to 2018. We stratified drugs into 3 competitiveness groups: drugs that are alone in their therapeutic class, those without generic equivalents but with competition from other distinct molecules in their class, and those with competition from identical generic competitors and from other distinct molecules in their class.

Finally, to illustrate out-of-pocket burden, we applied our framework to a hypothetical patient paying list prices at the pharmacy under a standard Part D plan. We estimated the effective out-of-pocket share that would have been paid by such a patient from 2014 to 2018. Effective out-of-pocket share for each drug and standard Part D benefit design phase was computed as defined in the equation presented previously. The coinsurance for branded prescription drugs in a standard Part D plan was 25% in the initial coverage phase and 5% in the catastrophic coverage phase throughout 2014 to 2018. The coinsurance for branded drugs in the coverage gap (ie, the donut hole) was 47.5%, 45%, 45%, 40%, and 35% in 2014, 2015, 2016, 2017, and 2018, respectively. We summarized the trends in the volume-weighted effective out-of-pocket share for drugs in our sample for the initial coverage phase, the coverage gap phase, and the catastrophic coverage phase, overall and by level of competition. Formulas appear in the eAppendix in the Supplement. We weighted the out-of-pocket obligation for each drug by the number of Part D units sold in the relevant year to construct out-of-pocket obligation for the average unit sold. Data analysis was conducted from June to December 2020 and conducted in Stata version 15 (StataCorp).

## Results

There were 3322 unique National Drug Codes (16 610 National Drug Code–years) in the analysis, representing 232 distinct molecules from 138 therapeutic classes in 34 disease areas (Table 1). From 2014 to 2018, the number of drugs in a multimolecule class in our sample increased from 3126 (94.1%) to 3170 (95.4%), and the number of drugs with generic equivalents increased from 1743 (52.5%) to 2116 (63.7%).

**Table 1.** Characteristics of Included NDCs, 2014-2018

Characteristic	Year, No.				
	2014	2015	2016	2017	2018
Disease areas	34	34	34	34	34
Therapeutic classes	138	138	138	138	138
Distinct molecules	232	232	232	232	232
Unique NDCs	3322	3322	3322	3322	3322
NDCs, No. (%)					
In a multimolecule class	3126 (94.1)	3134 (94.3)	3136 (94.4)	3160 (95.1)	3170 (95.4)
With generic equivalents	1743 (52.5)	1824 (54.9)	1889 (56.9)	1997 (60.1)	2116 (63.7)

Abbreviation: NDC, National Drug Code.

**Figure 1.**
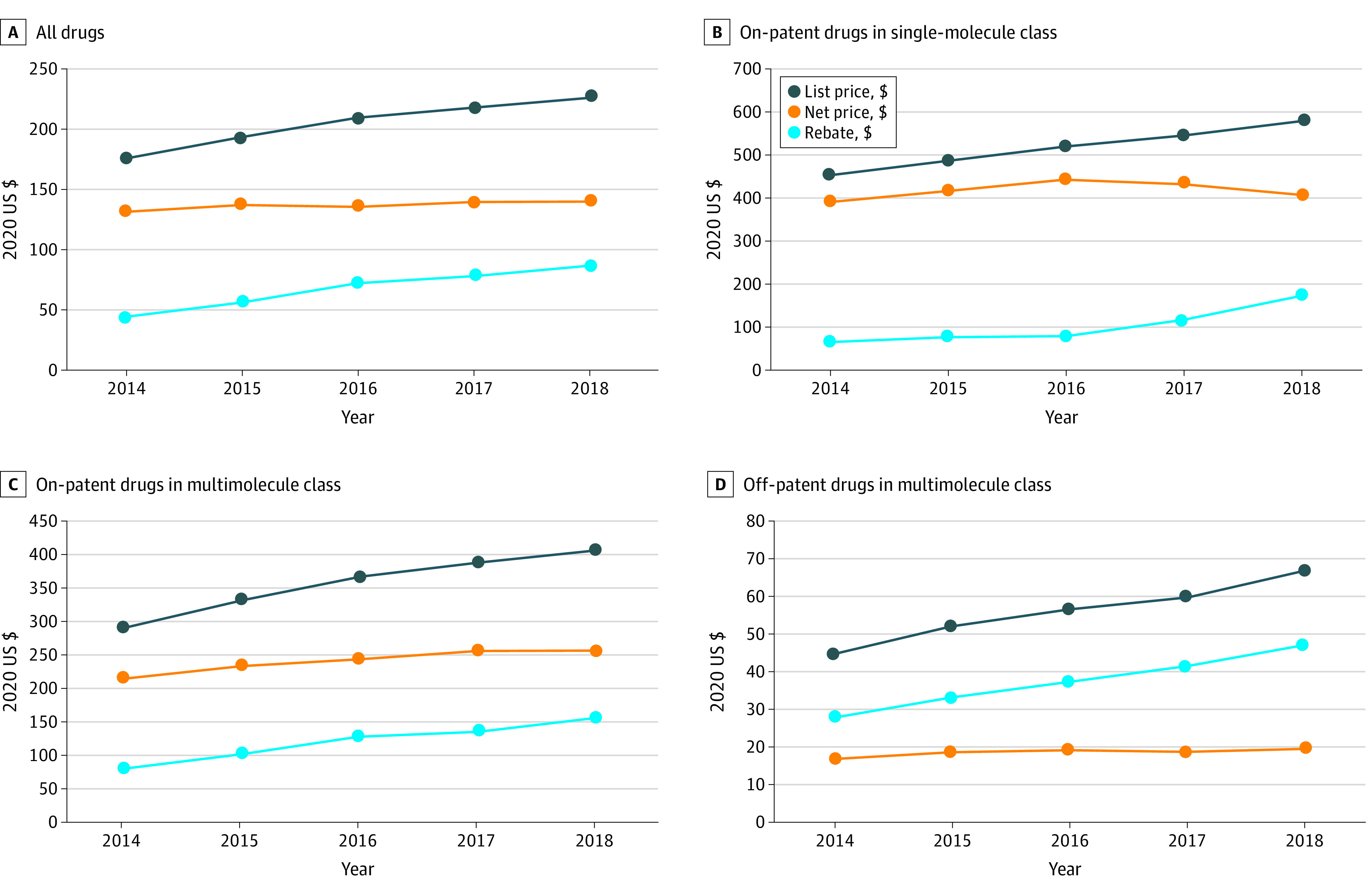
Trends in List Price per Unit, Net Price per Unit, and Rebate per Unit

**Figure 2.**
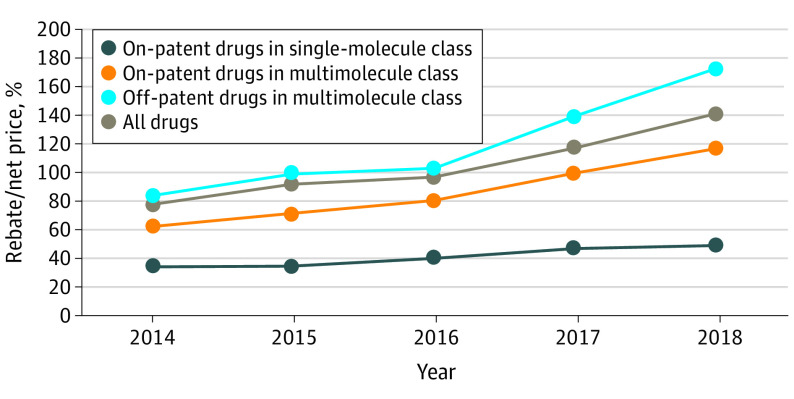
Trends in Rebate/Net Price From 2014 to 2018 for All Drugs and by Level of Competition

From 2014 to 2018, the mean (SD) list price per unit increased 29%, from $175 ($1438) to $226 ($1589), whereas mean (SD) net price increased 7%, from $131 ($1319) to $139 ($1186) (Figure 1). The growing divergence between list and net prices was consistent with the mean (SD) rebate, which increased from $44 ($243) to $87 ($520), a 98% increase. In this period, the mean list price was ere 32% higher than the net price in 2014 and 62% higher in 2018. The list-to-net ratio grew fastest for drugs with branded and generic competitors, from 2.7 in 2014 to 3.4 in 2018, compared with drugs with branded competitors only (from 1.4 to 1.6) and drugs without any competition (from 1.2 to 1.4). Table 2 displays the associated growth rates for the average drug, which exhibited annual list price growth of 10.1% (95% CI, 9.8%-10.3%), annual net price growth of 4.8% (95% CI, 4.4%-5.3%), and annual rebate growth of 17.3% (95% CI, 16.5%-18.0%). Growth rates were higher for the average drug than for the mean overall price, suggesting that price growth was higher for drugs with lower price levels (ie, the drugs with the lowest initial prices exhibited the fastest percentage growth). The same pattern appeared for net prices. The ratio of rebate to net price (ie, the retail price markup) increased from 77% to 140%, an annual increase of 15.2 percentage points or 11.9% (Figure 2).

**Table 2.** List Price, Net Price, Rebate, and Rebate/Net Price for Drugs With Different Levels of Brand-to-Brand and Generic Competition^a^

Annual increase	All (n = 3322)	On-patent drugs		Off-patent drugs in multimolecule class (n = 1480)
		Single-molecule class (n = 124)	Multimolecule class (n = 1046)	
**List price**					
Amount, $	12.5 (9.1 to 16.0)	30.9 (−0.61 to 62.4)	28.6 (19.1 to 38.1)	5.2 (3.5 to 6.9)
Growth, %	10.1 (9.8 to 10.3)	10.8 (9.9 to 11.7)	9.3 (8.9 to 9.6)	11.1 (10.7 to 11.5)
**Net price**					
Amount, $	1.8 (−0.83 to 4.4)	4.7 (−1.4 to 10.7)	10.1 (6.3 to 14.0)	0.58 (0.28 to 0.88)
Growth, %	4.8 (4.4 to 5.3)	7.4 (6.5 to 8.3)	2.9 (2.4 to 3.5)	4.5 (3.8 to 5.2)
**Rebate**					
Amount, $	10.8 (7.7 to 13.8)	26.2 (−9.7 to 62.2)	18.5 (12.0 to 24.9)	4.6 (3.0 to 6.3)
Growth, %	17.3 (16.5 to 18.0)	21.6 (16.9 to 26.4)	18.4 (17.4 to 19.3)	19.0 (17.8 to 20.3)
**Rebate/net price**					
Percentage point	15.2 (13.6 to 16.7)	5.8 (2.4 to 9.1)	13.8 (12.3 to 15.2)	21.9 (19.4 to 24.4)
Percentage	11.9 (10.9 to 12.8)	13.2 (8.7 to 17.9)	15.0 (13.8 to 16.2)	13.9 (12.4 to 15.3)

^a^ A total of 2650 drugs that did not change group between 2014 and 2018 were included. To estimate the annual absolute increase, list price, net price, rebate, and rebate/net price were regressed on calendar year, with fixed effects for each National Drug Code and standard errors clustered at the National Drug Code level. To estimate the annual percentage increase, the logarithm of list price, net price, rebate, and rebate/net price were regressed on calendar year, similarly with National Drug Code fixed effects and standard errors clustered at the National Drug Code level. All prices are expressed in 2020 US dollars.

When data were stratified by the level of competition, we observed similar patterns across all groups of drugs: between 2014 and 2018, list prices and rebates were increasing faster than net prices, and the ratio of rebates to net prices was also increasing (Figure 1B, 1C, and 1D and Figure 2). However, the degree of competition was associated with these outcomes. Figure 1 (panels A-C) shows that mean list price and net price were highest for the least competitive markets (ie, drugs without generic equivalents in single-molecule classes; mean [SD] list price, $520 [$2463]; mean [SD] net price, $418 [$1975]). Next highest were the classes with brand-to-brand competition only (mean [SD] list price, $355 [$1383]; mean [SD] net price, $237 [$983]), and the lowest were those with branded and generic competitors (mean [SD] list price, $56 [$395]; mean [SD] net price, $19 [$94]). Figure 2 shows the associated changes in the ratio of rebates to net prices (ie, the retail price markup). It was higher and increased faster for more competitive classes and vice versa (most competitive class, from 83% to 172%; next most competitive class, from 61% to 115%; least competitive class, from 33% to 49%).

**Figure 3.**
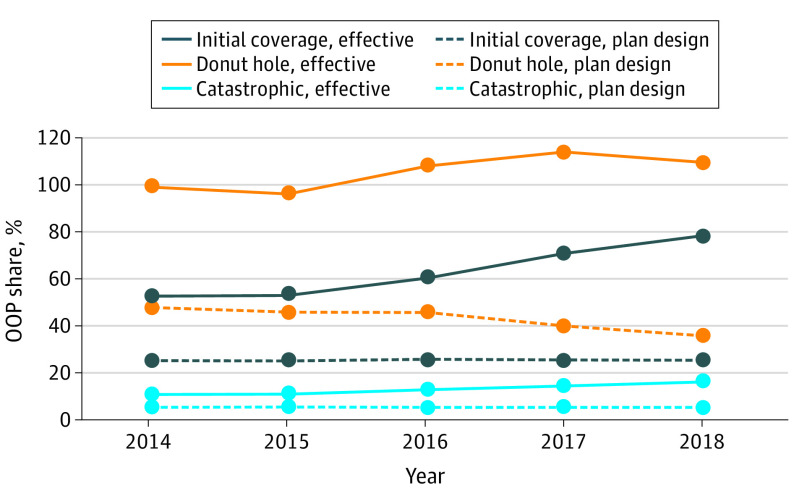
Patient’s Effective Out-of-Pocket (OOP) Share With a Standard Part D Plan From 2014 to 2018

For a patient paying list prices in a standard Part D plan, effective out-of-pocket share would have increased from 48% in 2014 to 64% in 2018 in the initial coverage phase, and from 10% to 13% in the catastrophic coverage phase, an increase of approximately 30% in both phases (Figure 3). Despite efforts to close the coverage gap, effective out-of-pocket share in the donut hole first increased from 92% in 2014 to 98% in 2016 and then decreased to 90% in 2018, barely below its 2014 level. Total Part D spending on drugs in our sample increased from $40.3 to $59.9 billion from 2014 to 2018, representing 49% to 60% of the total Part D spending in those years (eTable 1 in Supplement). Drugs with branded competition in our sample accounted for the largest share of Part D spending, from 26% in 2014 to 43% in 2018 (eTable 2 in Supplement).

These trends were magnified for drugs in the most competitive classes (eFigure in the Supplement). For every coverage phase, effective patient out-of-pocket cost share increased the most (in both percentage and percentage point terms) for drugs with branded and generic competitors, the next most for those with branded competitors, and the least for those without any competition. Compared with drugs with no competition, effective out-of-pocket share paid by patients grew 50% faster for drugs with branded competitors and 100% faster for those with branded and generic competitors. The numbers for the catastrophic coverage phase (eFigure in the Supplement) are instructive. The standard benefit specifies 5% cost sharing in this phase. By 2018, effective cost sharing ranged from 9% to 16% for the least to most competitive classes, respectively. Price levels were lower for drugs with generic competitors, mitigating the cost-sharing growth. However, patients paid more than twice the standard cost sharing for drugs with branded competitors and nearly twice for drugs with no competitors.

## Discussion

Using data on branded prescription drugs in the US from 2014 to 2018, we found that the average inflation-adjusted list price, net price, and rebate for drugs in our sample increased by 29%, 7%, and 98%, respectively, in 5 years. As did prior studies using SSR Health data, we found increasing divergence between list and net prices.^2^ The ratio of rebate to net price, a proxy for the retail price markup patients face at the point of sale, increased from 77% to 140%. Although mean list and net prices were lower in more competitive classes, retail price markups started from a higher initial level and increased faster in more competitive classes precisely because competition drives up rebate payments as a percentage of the price and on average exposes patients to higher point-of-sale prices and lower effective plan generosity. These patterns are consistent with economic theory, which suggests that pharmaceutical competition provides payers and pharmacy benefit managers with more leverage to seek higher rebates.^15^

For Medicare Part D patients with standard plan coinsurance obligations who were ineligible for the low-income subsidy, the effective out-of-pocket share increased from 48% to 64% in the initial coverage phase from 2014 to 2018 and from 10% to 13% in the catastrophic coverage phase. Despite efforts by policy makers to close the coverage gap, effective out-of-pocket share in the donut hole remained greater than 90% between 2014 and 2018. The disparity between effective out-of-pocket share and the coinsurance rate written in the benefit design was larger in more competitive markets.

Total Part D spending on drugs in our sample was $59.9 billion in 2018, accounting for approximately 60% of total Part D spending in that year (eTable 1 in the Supplement).^16^ Drugs with competition from branded competitors accounted for the largest share of spending in our sample, approximately 43% of the total Part D spending in 2018 (eTable 2 in the Supplement). For this group of drugs, consumers would pay more than double the statutory cost sharing under a standard Part D benefit design when exposed to list prices. Typically, competition helps consumers. However, exposing enrollees to list price–based coinsurance inverts this conventional logic. Absent reform, attempts to reduce the out-of-pocket burden for patients may be frustrated by competition that leads to higher rebates and retail prices, which implicitly trade away generous coverage in exchange for lower premiums, shifting the burden from taxpayers and premium-paying beneficiaries to patients. Out-of-pocket burden creates more financial risk from drug spending for patients, and higher out-of-pocket cost is associated with lower rates of initiation of drug treatment, worse adherence, and more frequent discontinuation of therapy.^17^

In principle, there is a way out of this dilemma. Starting in 2019, some insurers began to offer their commercial customers the option to include point-of-sale rebate pass-through with their plans.^18,19^ If taken up, these options would pass the rebate payment through to the consumer at the point of sale. The pass-through would reduce the consumer’s out-of-pocket obligation by ensuring that coinsurance applies only to the net price. Prior research estimated that basing cost sharing on net price, or full pass-through of rebate at point of sale, would reduce out-of-pocket spending for nearly half of Part D beneficiaries who do not receive low-income subsidies.^20^ Approximately 20% of these beneficiaries would save more than $100 per year, and approximately 1% would save more than $1000 per year.^20^

Under these arrangements, confidentiality could still be protected by, for example, bundling rebates of several drugs during the course of the year, making it hard or even impossible to infer the precise rebate paid for each drug.^21,22^ However, adoption of point-of-sale rebate plans has been slow. By the end of 2019, only 20% of large employers included point-of-sale rebates in their pharmacy plans.^23^ Furthermore, there are currently no provisions for point-of-sale rebates in the Medicare Part D program, despite interest by some policy makers in the concept.^24^

Why has uptake been slow? Payers may be deterred by possible effects on premiums. Prior research found that full pass-through of rebates at point of sale would result in a 13% increase in Part D premiums.^25^ Some beneficiaries would have premium increases that more than offset their reduced out-of-pocket spending. In addition, because pharmacy benefit managers do not receive rebates from manufacturers until weeks or months after a drug is dispensed, the point-of-sale rebate to the consumer may not be equal to the insurer’s true rebate revenue. Payers must choose a method to project what the rebate will be.^22^

### Limitations

This study has several limitations. First, the sample of drugs included in the data set was limited to branded products marketed by publicly traded companies. Second, in the data set, net prices accounted for all manufacturer concessions, not just rebates. Nonetheless, these indirect estimates of net price at the product level are, to our knowledge, the only commercially or publicly available source of information on product-level net prices in the marketplace. Third, in reality, trends in retail price markup and effective insurance generosity are not experienced equally across insurers, patients, or drugs. Pharmacy benefit managers negotiate different rebates for different clients and different drugs; the benefit design of an actual Part D plan may deviate from that of the standard plan; retail pharmacy prices may not always relate to list prices; and patients may reduce their out-of-pocket spending through patient assistance programs. Additionally, payers such as Medicaid and the Veterans Administration can extract larger rebates or discounts than commercial payers primarily as a result of regulation.^26,27^ We were not able to quantify this variation in excess patient burden across payers with our data, but it remains relevant to consumer welfare. Fourth, we used whether a drug has a generic equivalent and whether there are other molecules in the same therapeutic class to measure generic and branded competition. However, there may be competition from molecules from other therapeutic classes for the same condition. Fifth, the total dosage unit reported by the Medicare Part D Drug Spending Dashboard reflects multiple routes of administration of the drug (eg, intravenous, subcutaneous) and multiple strengths, which may feature different pricing in the data set. Our volume-weighted out-of-pocket share estimates assumed equal share of total dosage across routes of administration and strengths.

## Conclusions

Policy makers are increasingly recognizing the role of rebates in driving consumer cost sharing. Our study illustrated how they also invert the effects of competition on patient welfare. Under the current rebate system, greater competition may increase cost-sharing burdens for patients paying coinsurance. Payers passing rebates through to consumers at the point of sale could help restore the benefits of pharmaceutical competition and rebates to patients.

## Notes

Supplement. **eAppendix.** Estimating Volume-Weighted Effective Out-of-Pocket Cost of Prescription Drugs in Medicare Part D **eTable 1.** Part D Spending on Drugs in Our Sample **eTable 2.** Part D Spending on Drugs by Level of Competition **eFigure.** Patient’s Effective Out-of-Pocket Share With a Standard Part D Plan From 2014 to 2018 for Drugs With Different Levels of Competition
