# Supplementary material for: Association of Drug Rebates and Competition With Out-of-Pocket Coinsurance in Medicare Part D, 2014 to 2018
Source: JAMA Netw Open. 2021 May 5;4(5):e219030. doi: 10.1001/jamanetworkopen.2021.9030 (PMC8100863; doi:10.1001/jamanetworkopen.2021.9030)
Supplement: Supplement. — eAppendix. Estimating Volume-Weighted Effective Out-of-Pocket Cost of Prescription Drugs in Medicare Part D eTable 1. Part D Spending on Drugs in Our Sample eTable 2. Part D Spending on Drugs by Level of Competition eFigure. Patient’s Effective Out-of-Pocket Share With a Standard Part D Plan From 2014 to 2018 for Drugs With Different Levels of Competition [file jamanetwopen-e219030-s001.pdf]

Supplemental Online Content

Lakdawalla D, Li M. Association of drug rebates and competition with out-of-pocket coinsurance in Medicare Part D, 2014-2018. *JAMA Netw Open*. 2021;4(5):e219030. doi:10.1001/jamanetworkopen.2021.9030

eAppendix. Estimating Volume-Weighted Effective Out-of-Pocket Cost of Prescription Drugs in Medicare Part D

eTable 1. Part D Spending on Drugs in Our Sample

eTable 2. Part D Spending on Drugs by Level of Competition

eFigure. Patient's Effective Out-of-Pocket Share With a Standard Part D Plan From 2014 to 2018 for Drugs With Different Levels of Competition

This supplemental material has been provided by the authors to give readers additional information about their work.

© 2021 Lakdawalla et al. *JAMA Network Open.*

eAppendix. Estimating volume-weighted effective out-of-pocket (OOP) cost of prescription drugs in Medicare Part D

The coinsurance rate in the initial coverage phase for a standard Part D plan is 25%, the volume-weighted effective OOP cost for drug d is:

$$Effective\ OOP\ Share_d = w_d 25\% \left(1 + \frac{Rebate_d}{Net_d}\right)$$

$$w_d = \frac{v_d}{V}$$

where v_d is the total units sold in Part D for drug d, and V is the total units sold for all drugs in our sample in Part D.

For the coverage gap and the catastrophic coverage phase, the coinsurance rate is different, but the rest of the calculation is the same.

© 2021 Lakdawalla et al. *JAMA Network Open.*

eTable 1. Part D spending on drugs in our sample

Year	Total part D spending on drugs in our sample, billion	Total part D spending, billion [a]	% of total part D spending
2014	40.3	81.8	49.3
2015	47.5	89.6	53.0
2016	52.7	92.7	56.8
2017	55.6	93.9	59.2
2018	59.9	99.5	60.2

[a] Source: KFF 10 Essential Facts About Medicare and Prescription Drug Spending. Published Jan 29, 2019.

eTable 2. Part D spending on drugs by level of competition

Year	Number of products	Total part D spending on these products, billion	% of total Part D spending
Drugs with no competition			
2014	42	1.9	2.3
2015	42	2.5	2.8
2016	42	3.3	3.6
2017	42	4.1	4.4
2018	42	5.0	5.0
Drugs with brand-to-brand competition			
2014	296	21.5	26.3
2015	297	26.9	30.0
2016	296	31.7	34.2
2017	299	36.8	39.2
2018	298	42.9	43.1
Drugs with brand-to-brand and generic competition			
2014	221	3.7	4.6
2015	220	4.1	4.6
2016	221	4.4	4.7
2017	218	4.4	4.7
2018	219	4.4	4.5

Note. Drugs with no competition are those in a single-molecule class without generic equivalents. Drugs with brand-to-brand competition are those in a multi-molecule class but do not have generic equivalents. Drugs with brand-to-brand and generic competition are those in a multi-molecule class and have generic equivalents.

© 2021 Lakdawalla et al. *JAMA Network Open.*

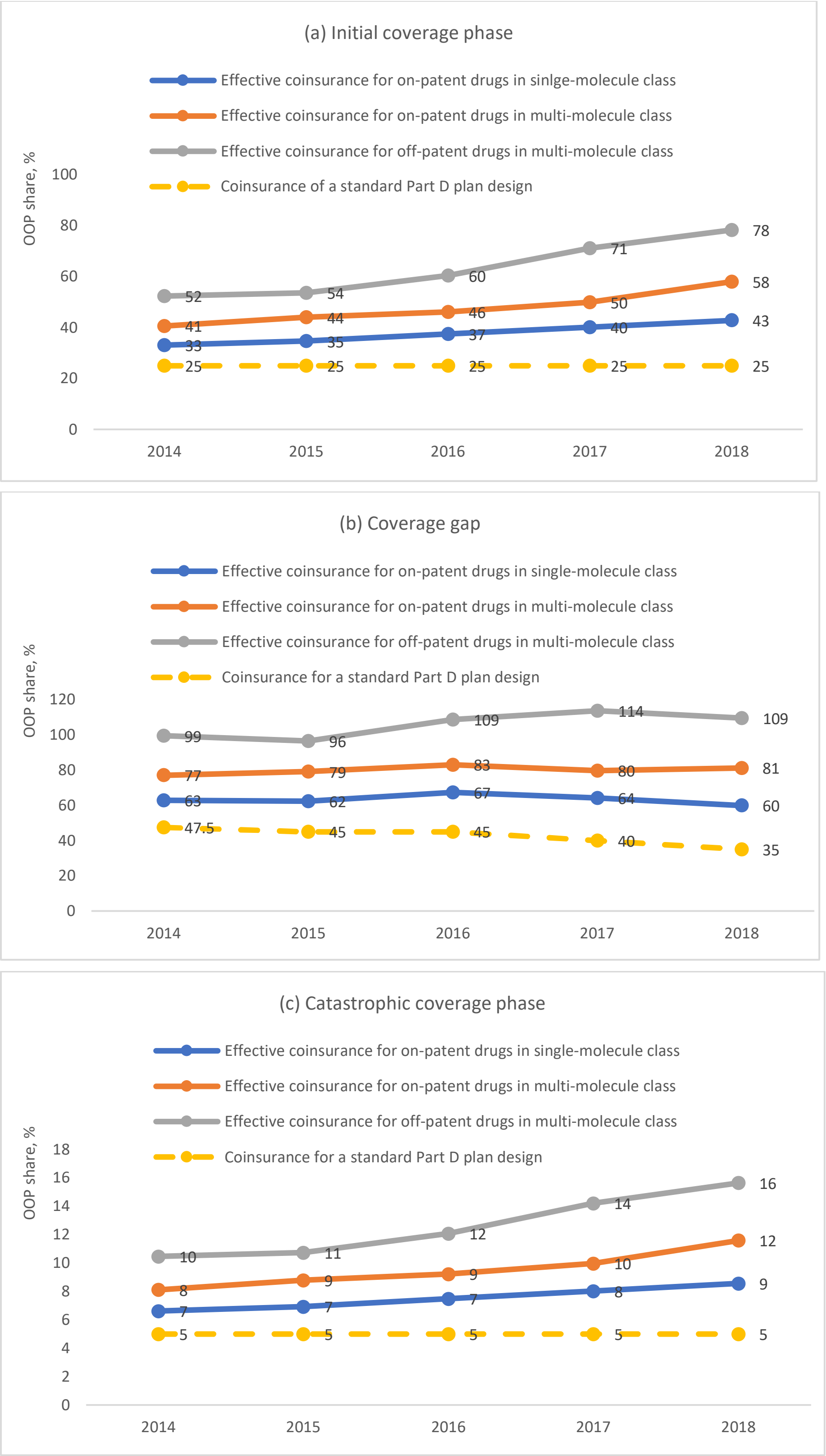

eFigure. Patient's effective out-of-pocket share with a standard Part D plan from 2014 to 2018 for drugs with different levels of competition

© 2021 Lakdawalla et al. *JAMA Network Open.*
